# The Prognostic Value of Tumor Size, Volume and Tumor Volume Reduction Rate During Concurrent Chemoradiotherapy in Patients With Cervical Cancer

**DOI:** 10.3389/fonc.2022.934110

**Published:** 2022-07-14

**Authors:** Chang Sun, Shubin Wang, Wenjing Ye, RanLin Wang, Mingyu Tan, Hanyi Zhang, Jie Zhou, Minglun Li, Lichun Wei, Peng Xu, Guiquan Zhu, Jinyi Lang, Shun Lu

**Affiliations:** ^1^ Department of Radiation Oncology, Sichuan Cancer Hospital & Institute, Sichuan Cancer Prevention and Control Center, School of Medicine, University of Electronic Science and Technology of China, Chengdu, China; ^2^ School of Medicine, University of Electronic Science and Technology of China, Chengdu, China; ^3^ Department of Radiation Oncology, University Hospital, Ludwig-Maximilians-Universität München (LMU), Munich, Germany; ^4^ Department of Radiation Oncology, Xijing Hospital, Air Force Medical University, Xi’an, China; ^5^ State Key Laboratory of Oral Diseases, National Clinical Research Centre for Oral Diseases, Department of Head and Neck Oncology, West China Hospital of Stomatology, Sichuan University, Chengdu, China; ^6^ Radiation Oncology Key Laboratory of Sichuan Province, Chengdu, China

**Keywords:** cervical cancer, radiation therapy, tumor parameters, prognostic factors, tumor volume reduction rate (TVRR)

## Abstract

**Objective:**

This study aimed to investigate the relationship between prognostic and tumor parameters of cervical cancer patients, such as tumor size (TS), tumor volume (TV), and tumor volume reduction rate (TVRR) after external beam radiotherapy.

**Methods:**

A total of 217 patients with advanced cervical cancer, classified as Federation of Gynecology and Obstetrics (FIGO) IIa–IVa, were enrolled in the study. Pre- and mid-RT pelvic magnetic resonance imaging (MRI) were performed twice, during RT and just before brachytherapy.

**Results:**

The median follow-up time was 51 months (range, 7–111 months). The 5-year overall survival (OS), progression-free survival (PFS), and local failure-free survival (LFFS) rates were 81.3, 85.1, and 92.9%, respectively. Multivariate analysis revealed that tumor parameters including FIGO stage >II (Hazard Ratio, 2.377 and 95% confidence interval [CI], 1.091–5.182; *P* = 0.029), pre-RT TV >61.6 cm^3^ (HR, 0.417 and 95% CI, 0.188–0.926; *P* = 0.032), and mid-RT TV >11.38 cm^3^ (HR, 3.192 and 95% CI, 1.094–9.316; *P* = 0.034) were observably associated with OS. Univariate analysis showed that the tumor volume reduction rate (TVRR) was dramatically associated with overall survival (HR, 0.204 and 95% CI 0.033–1.282; *P <*0.001) and local failure-free survival (*P* = 0.050).

**Conclusions:**

In this retrospective study, TVRR and mid-radiotherapy tumor volume are independent and strong prognostic parameters for patients with local advanced cervical cancer receiving CCRT.

## Introduction

Cervical cancer is the 4th most common cause of cancer incidence and mortality in women worldwide, severely endangering the health of women in developing countries (1). The mainstay treatment of advanced cervical cancer is the combination of concurrent chemotherapy with external beam radiation therapy (EBRT) followed by an intracavitary brachytherapy (ICBT) boost (2–4), and the external beam with intensity modulated simultaneous integrated boost can be used to replace brachytherapy in selected patients who cannot undergo intracavitary radiation treatment (5). Previous studies demonstrated that survival outcomes of cervical cancer patients could be predicted by lymph node (LN) status, primary tumor size (TS), FIGO stage, age, and histology (6–8). In particular, the volume of residual tumor after radiotherapy (RT) is associated with therapeutic effect, which can be assessed readily by MRI (9, 10). Furthermore, it has been reported that MRI could provide a better evaluation value than some biomarkers for the treatment response in locally advanced cervical cancer (11). Currently, it is also difficult to guide the prognosis evaluation of patients and stratify patients with persistent or recurrent disease into risk groups because of the heterogeneity of the associated factors and the radiosensitivity (12). However, most previous studies have reported these tumor prognostic factors with respect to survival outcomes after the completion of RT (7, 13). Although the response after completion of RT is important, we hypothesized that tumor parameters, such as tumor size (TS), tumor volume (TV), and tumor volume reduction rate (TVRR), measured before the intracavitary radiotherapy (ICR), appear to have greater prognostic value (10, 12, 14). It might also optimize treatment regimens at an appropriate time based on the earlier response evaluation (15–18). Thus, our study aimed to demonstrate the prognostic significance of these tumor parameters during treatment in 217 cervical cancer patients treated with concurrent chemoradiotherapy (CCRT) and adequate follow-up were reviewed.

## Materials and Methods

### Patient Population

We retrospectively reviewed 217 patients with cervical cancer who received definitive CCRT in the Sichuan cancer hospital from April 2009 to November 2016, and had no prior history of radiation therapy to the pelvis. All patients had histologically confirmed squamous cell carcinoma of the cervix. Additionally, their cancers were classified as 2018 FIGO stages IIa–IVa by initial clinical examination under local anesthesia, chest and abdominopelvic computed tomography (CT), and pelvic magnetic resonance imaging (MRI). We excluded patients who had a history of other histologically confirmed cancers, those who had recurrent cancer or distant metastasis, those who had no measurable tumor on the MRI both pre-RT and during RT (before brachytherapy), and those who were treated with neoadjuvant chemotherapy or had incomplete treatment.

### Treatment

#### Concurrent Chemotherapy and External Beam Radiotherapy (EBRT)

Candidates were given a combination of EBRT and ICBT treatment. The EBRT was integrated with an intensity-modulated radiotherapy (IMRT) treatment planning system (Oncentra TOMO and Pinnacle) and delivered using a dynamic multi-leaf linear accelerator with photon energy of 10 MV. Full bladder during simulation and irradiation was required to minimize the small bowel volume in the target volume. The gross tumor volume (GTV) was defined as the cervix tumor. GTV-N was defined as positive nodes. The cervix clinical target volume should include the GTV, cervix (if not already encompassed by the GTV), uterus, parametria, ovaries, and vaginal tissues. The nodal clinic tumor volume (CTV) must include involved nodes and relevant draining nodal groups (common, internal, and external iliacand, obturator and presacral lymph nodes). Inclusion of para-aortic lymph nodes will depend on the extent of disease and the results of staging investigations. The planning target volume nodal CTV and GTV-N was created by creating a 5–7 mm margin around the CTV nodal and GTV-N. The PTV cervix was created by performing a 5–7 mm geometric expansion of the CTV cervix. Prior to BT, patients were treated with 45 Gy (25 fractions of 1.8 Gy) EBRT using an IMRT technique with 10 MV photons. Some patients with advanced bulky disease received 50.4 Gy (28 fractions of 1.8 Gy). Cncomitant IMRT boost to 54–60 Gy to the positive lymph nodes to encompass at least 95% volume.

During radiotherapy, all patients received a concurrent chemotherapy regimen based on cisplatin every three weeks (80–100 mg/m^2^). Dose modifications were prescribed for subsequent cycles based on toxicity grade (4). The tumor shape was defined as the location where the tumor grows in the pelvic cavity, the eccentric was defined as whether the tumor center deviates more than 2 cm from the center axis of the pelvic cavity, and the center was defined as whether the tumor center deviates less than 2 cm from the center axis of the pelvic cavity. Acute toxicity reactions to CCRT were assessed weekly using the Common Terminology Criteria for Adverse Events (CTCAE) v4.0.

#### Brachytherapy

The tumors were given a combination treatment of EBRT and brachytherapy (19, 20), delivered in 4–5 fractions, twice a week, of approximately 6–7 Gy per fraction. All patients underwent CT-based planning with custom immobilization and the contrast-enhanced CT scan images were obtained using a radiopaque marker to define the cervix and upper vagina before contouring. According to GEC-ESTRO recommendations, GTV, high-risk CTV (HR-CTV), and intermediate risk CTV (IR-CTV) were identified from the fusion of CT and MRI images that used the image registration of the applicator. GTV is defined as a cervix tumor. HR-CTV included the entire cervix and the presumed extracervical tumor extension at the time of BT. On the basis of HR-CTV, IR-CTV was limited by the natural anatomical borders of the rectal and bladder wall. A safety margin of up to 3–5 mm was taken, in the anterior–posterior direction, 5–10 mm was used in the superior–inferior direction, and 5–10 mm was applied to both parametrics, in the lateral direction (19). The OARs include the bladder, rectum, sigmoid colon, and small intestine (19, 20). The total cumulative dose of EBRT and brachytherapy boost was evaluated in terms of equivalent dose in 2 Gy per fraction (EQD2), using α/β = 3 Gy for OAR and α/β = 10 Gy for targets. The treatment planning aimed to achieve D90 >86 Gy for HR-CTV and D90 >75 Gy for IR-CTV from combined EBRT and brachytherapy. Dose volume constraints for the cumulative dose to the OAR were D2cc <90 Gy for the bladder, and D2cc <75 Gy EQD2 for the rectum and sigmoid.

## Study Procedures

Generally, tumor volumes are classified on the basis of initial disease burden (quantified by pre-RT TV and pre-RT TS) and response to CCRT in the treatment course. Treatment response was evaluated by comparing the ratio of mid-RT TV to pre-RT TV. The original study protocol encompassed two assessment sessions of the same patient, including pre-RT MRI performed within 2 weeks before the start of EBRT (‘baseline evaluation’) and mid-RT MRI within 1 week before starting brachytherapy (‘mid-term evaluation’). Tumor size on MRI was imaged using an axial T2-weighted sequence with 1.5 T or 3.0 T MRI. For the primary tumor size (TS), defined as the maximum width on axial T2-weighted sequences. Two radiation oncologists defined the tumor areas of each slice. The primary tumor volume (TV) was calculated by the summation of all tumor areas for each slice of the MRIs and multiplication by the profile of slices. All participating radiation oncologists generally comply with RTOG guidelines (21). We also recommended that it would be better to have another experienced radiologist to review the contouring process and to be responsible for the quality control. Finally, two tumor volumes for each patient were obtained: pre-RT tumor volume (V1) and mid-RT tumor volume (V2). The TVRR was defined as the percentage of TV that is significantly reduced on the mid-RT MRI scan relative to the pre-RT MRI scan. Tumor volume reduction rate (TVRR): TVRR = (V1 − V2/V1) × 100% (22).

### Follow-Up

Patients were assessed for disease-related parameters and adverse side effects according to institutional guidelines at regular intervals. In general, this was every 3 months in the first 2 years, every 6 months for the next 3 years, and then annually thereafter. An MRI was done every 6 months in the first 5 years. Post-treatment evaluation included physical examination, laboratory studies, and radiological work-up, such as MRI, CT, or other examination if necessary, to assess and document treatment outcome as well as complications. We classified failures according to the site of recurrence as follows: local failure was defined as recurrence at the cervix or vagina; and distant metastasis (DM) was defined as visceral organ or non-pelvic lymph node metastasis, including the para-aortic lymph node and inguinal lymph node. Time intervals for OS, LFFS, and PFS were calculated from the date of diagnosis to the date of event or last follow-up appointment.

### Statistical Analysis

The SPSS software (version 19, SPSS Inc., Chicago, IL, USA) was used for the statistical analysis. *T*-tests were used to analyze changes in tumor parameters (TS and TV) between the pre- and mid-RT points. OS and PFS were estimated with the Kaplan–Meier method. Prognostic factors (age, FIGO stage, RT dose, pre- and mid-RT TS and TV, and TVRR) were analyzed using the log-rank test and Cox regression model. To identify the optimal cut-off point of continuous tumor parameters with respect to a prognostic impact, we calculated the Youden index of the receiver operating characteristic (ROC) curve (14). We defined *p <*0.05 as statistically significant.

## Results

### Demographic Features

A total of 217 patients were registered, and 3 patients were eventually lost to follow-up (no recurrence or metastasis occurred by the last follow-up). The median follow-up for all investigated patients was 51 months (range 7–111 months). According to the 2018 version of FIGO stage distribution, IIa and IIb in 91 patients (41.9%); I in 114 patients (52.5%); and IVa in 12 patients (5.6%), as shown in **Table 1**. During the period of CCRT, a total of 207 patients (95.4%) showed complete response and 9 patients (4.1%) showed partial response in the short-term efficacy assessment (within 3 months of the full treatment of the bunching), with no disease progression.

**Table 1:** Patient characteristics.

Characteristics	Group	No. of patients (%)
FIGO stage	IIa, b	91 (41.9)
	III	114 (52.5)
	IVa	12 (5.6)
Tumor shape	eccentric	149 (68.7)
	center	68 (31.3)
Pre-RT TS	≤5.5 cm	143 (65.9)
	>5.5 cm	74 (34.1)
Pre-RT TV	≤61.6 cm^3^	61 (28.1)
	>61.6 cm^3^	156 (71.9)
Mid-RT TS	≤2.1 cm	55 (25.3)
	>2.1 cm	162 (74.7)
Mid-RT TV	≤11.38 cm^3^	107 (49.3)
	>11.38 cm^3^	110 (50.7)
TVRR	≤82.19%	81 (59.6)
	>82.19%	136 (40.4)

FIGO, International Federation of Gynecology and Obstetrics; RT, radiotherapy; TS, tumor size; TV, tumor volume; TVRR: tumor volume reduction rate.

### Tumor Parameter Ranges Before and After EBRT

In all 217 patients, the median pre- and mid-RT maximum TS were 5.2 cm (range, 1.6–9.9 cm) and 2.8 cm (range, 0–6.3 cm), respectively (*P* <0.001). The median pre- and mid-RT TV were 95.9 cm^3^ (range, 3.9–560 cm^3^) and 11.5 cm^3^ (range, 0–99.1 cm^3^), respectively (*P* <0.001).The median TVRR was 86.7% (range, 16.9–100%).

### ROC Correlation Analysis

The Youden index points in the ROC curve were used to determine the data cut-off points with the best sensitivity and specificity among continuous tumor parameters, and the corresponding data results are shown in **Table 2**.

**Table 2:** ROC relevant parameters and cut-off values.

	Youden index	sensitivity	specificity	cut-off values	AUC	*P*-value
Age	0.09685	51.35	58.33	47	0.523	0.674
Pre-RT TS	0.1428	45.95	68.33	5.5 cm	0.532	0.574
Pre-RT TV	0.1173	37.84	73.89	61.6 cm^3^	0.523	0.673
Mid-RT TS	0.07748	81.08	26.67	2.1 cm	0.506	0.896
Mid-RT TV	0.4308	89.19	53.89	11.38 cm^3^	0.696	<0.001
TVRR	0.4371	86.50	57.20	82.19%	0.712	<0.001

ROC, receive operating characteristic; AUC, area under curve; RT, radiotherapy; TS, tumor size; TV, tumor volume; TVRR, tumor volume reduction rate.

### Patterns of Failure

The follow-up period ended in August 2018. Three patients were lost to follow-up and the median follow-up time was 51 months (range, 5–11 months).During the follow-up period, a total of 31 patients (14.3%) experienced tumor progression after treatment, among whom 13 patients (6%) had LF and 18 patients (8.7%) had DM (18). The range, mean ± standard deviation, and changes of tumor parameters before and after EBRT are shown in **Table 3**.

**Table 3:** The range of tumor parameters and the changes before and after EBRT.

	Median	Range	Mean	Standard Deviation	P-Value
Pre-TS (cm)	5.2	1.6–9.9	5.39	1.11	P <0.001
Mid-TS (cm)	2.8	0–6.3	2.88	0.94	
Pre-TV (cm^3^)	95.9	3.9–540.0	112.27	76.87	P <0.001
Mid-TV (cm^3^)	11.5	0–99.1	18.76	19.44	
TVRR (%)	86.7	16.9–100	81	17	–

EBRT, external beam radiotherapy; SD, standard deviation; TS, tumor size; TV, tumor volume; TVRR, tumor volume reduction rate.

### Prognostic Factor Analysis for Survival

**Figure 1:**
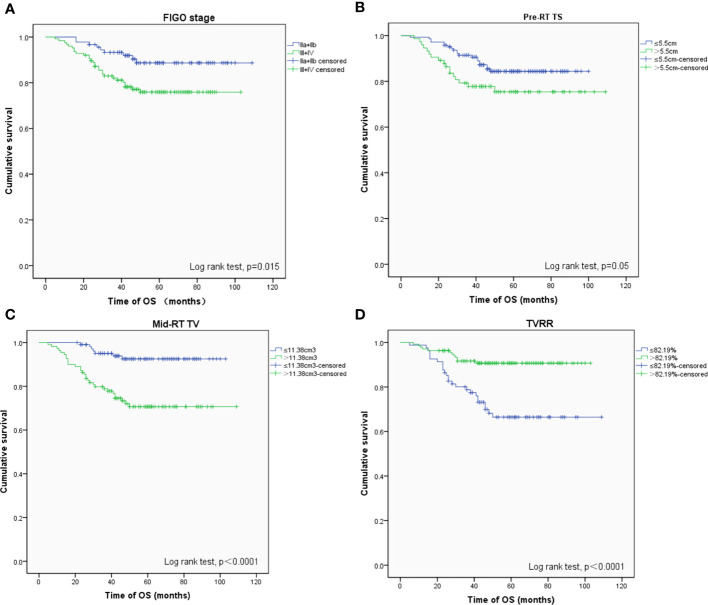
OS-related prognostic factors: **(A)** FIGO stage (phase II vs III+IV), **(B)** maximum diameter of tumors before radiotherapy (≤ 5.5 cm vs > 5.5 cm), **(C)** volume of tumors after external irradiation (≤ 11.38 cm 3 vs > 11.38 cm 3), **(D)** reduction rate of tumors (≤ 82.19% vs > 82.19%).

The 5-year OS, PFS, and LFFS rates were 81.3, 85.1, and 92.9%, respectively. A Cox univariate regression analysis was performed to evaluate the endpoint of OS. The variables analyzed were FIGO stage >II (*P* = 0.015), pre-RT TS >5. 5 cm (*P* = 0.050), mid-RT TV >11. 38 cm^3^ (*P <*0.001), and TVRR ≤82.19% (*P <*0.001) (**Figure 1** ). Factors associated with p-values <0.1 in the univariate analysis were included in the multivariate analysis. The variables that were found to be of independent prognostic significance for OS by multivariate analysis were FIGO stage >II (*P* = 0.029), pre-RT TV >61.6 cm^3^ (*P* = 0.032), and mid-RT TV >11.38 cm^3^ (*P* = 0.034) (**Table 4**).

**Table 4:** Prognostic analysis of overall survival.

Variable	No.	Univariate	*p*-Value	Multivariate	*p*-Value
		5-yr OS (%)		HR (95% CI)	
Age (yr)			0.236		0.155
≤47	94	78.4		1.633 (0.831–3.209)	
>47	123	83.7			
FIGO stage			0.015		0.029
IIa,IIb	91	88.7		2.377 (1.091–5.182)	
III,IV	126	75.9			
Pre-RT TS (cm)			0.050		0.154
≤5.5	143	84.4		0.578 (0.272–1.228)	
>5.5	74	75.4			
Pre-RT TV (cm^3^)			0.217		0.032
≤61.6	61	75.7		0.417 (0.188–0.926)	
>61.6	156	83.7			
Mid-RT TS (cm)			0.314		0.373
≤2.1	55	86.4		0.683 (0.295–1.581)	
>2.1	162	79.5			
Mid-RT TV (cm^3^)			<0.001		0.034
≤11.38	107	92.5		3.192 (1.094–9.316)	
>11.38	110	70.7			
TVRR (%)			<0.001		0.268
≤82.19	81	66.4		0.603 (0.247–1.474)	
>82.19	136	90.7			
Tumor shape			0.363		0.058
eccentric	149	82.8		1.930 (0.977–3.811)	
center	68	78			

OS, overall survival; HR, Hazard Ratio; CI, confidence interval; FIGO, International Federation of Gynecology and Obstetrics; RT, radiotherapy; TS, tumor size; TV, tumor volume; TVRR, tumor volume reduction rate.

**Figure 2:**
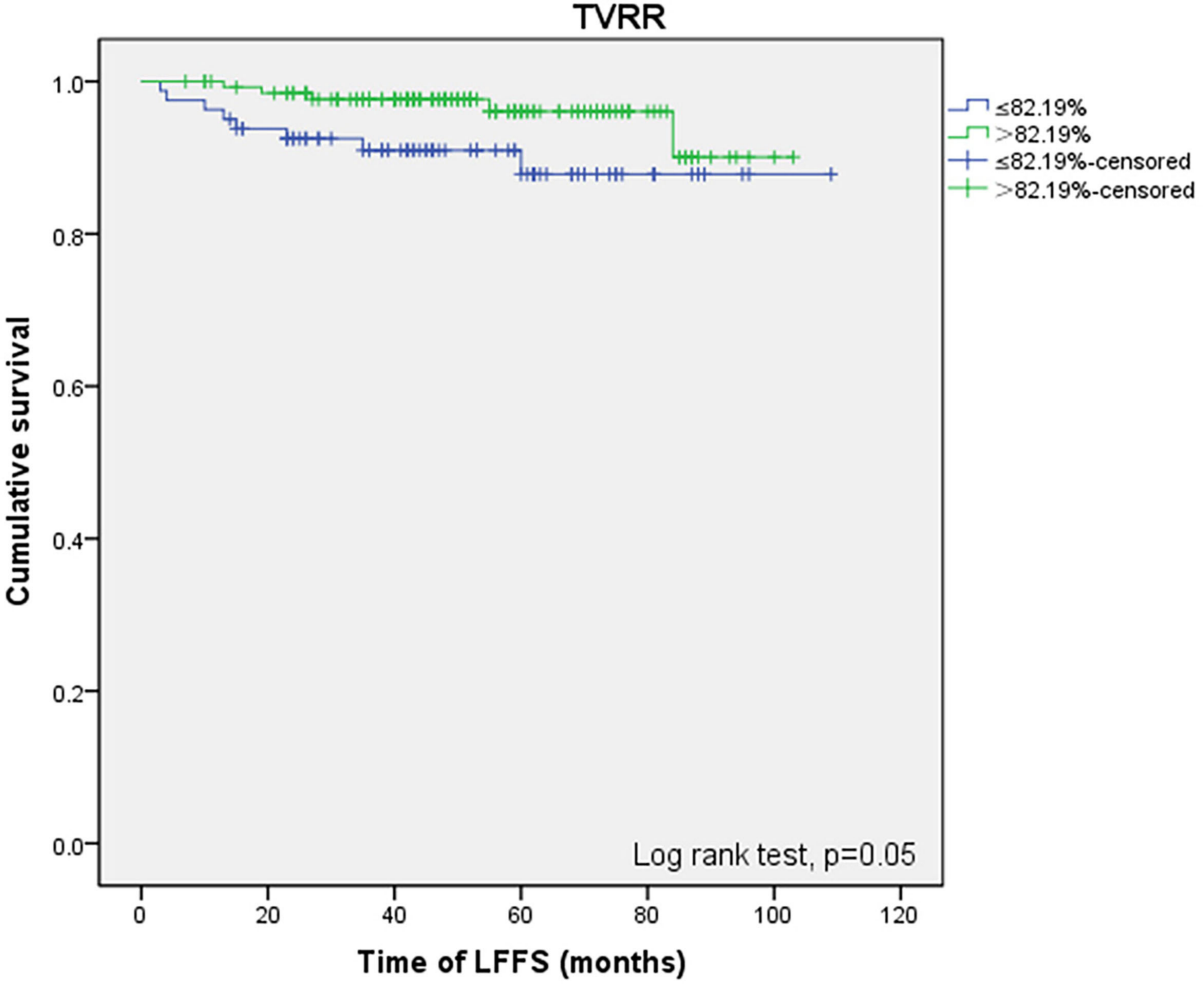
LFFS-related prognostic factors: tumor reduction rate (≤ 82.19% vs > 82.19%).

No positive results were obtained from the prognostic factors associated with PFS. As shown in **Table 5**, univariate analysis related to LFFS showed that TVRR ≤82.19% (*P* = 0. 050) (**Figure 2**) was an important factor for poor prognosis.

**Table 5:** Prognostic analysis of local failure-free survival.

Variable	No.	Univariate	*p*-Value	Multivariate	*p*-Value
		5-yr LFFS (%)		HR (95% CI)	
Age (yr)			0.161		0.391
≤47	94	89		1.692 (0.509–5.630)	
>47	123	95.7			
FIGO stage			0.518		0.505
IIa,IIb	91	91.6		1.490 (0.462–4.810)	
III,IV	126	94			
Pre-RT TS (cm)			0.099		0.051
≤5.5	143	94.6		0.289 (0.083–1.007)	
>5.5	74	89.1			
Pre-RT TV (cm^3^)			0.860		0.601
≤61.6	61	95.1		1.437 (0.370–5.586)	
>61.6	156	92			
Mid-RT TS (cm)			0.789		0.867
≤2.1	55	92.9		0.894 (0.240–3.330)	
>2.1	162	92			
Mid-RT TV (cm^3^)			0.332		0.450
≤11.38	107	96.2		2.059 (0.316–13.4370	
>11.38	110	89.6			
TVRR (%)			0.050		0.090
≤82.19	81	87.8		0.204 (0.033–1.282)	
>82.19	136	96.1			
Tumor shape			0.269		0.184
eccentric	149	95		2.211 (0.686–7.131)	
center	68	88.6			

LFFS, local failure-free survival; HR, Hazard Ratio; CI, confidence interval; FIGO, International Federation of Gynecology and Obstetrics; RT, radio-therapy; TS, tumor size; TV, tumor volume; TVRR, tumor volume reduction rate.

## Discussion

It is generally accepted that the outcome of patients with carcinoma of the uterine cervix is affected by several tumor-related variables, which include FIGO stage, immunohistochemical markers, blood indicators, etc. However, these indicators are not definitive criteria for determining which patients respond to CCRT and which patients need further treatment. Confirming worse survival patients during treatment by the response to CCRT may be predicted earlier and alternative initial treatment schedule (23, 24). Therefore, some indicators that can be measured by MRI easily and non-invasively during the treatment, such as TVRR, have received increasing attention from researchers. To find clinically meaningful predictors of the survival after CCRT. In this study, we tried to find clinically meaningful predictors of treatment reaction parameter TVRR, which has been reported in previous studies. This prognostic factor also reflects the early treatment response and radiation sensitivity, which might help formulate the optimal treatment strategy for cervical cancer patients during radiotherapy.

MRI is able to provide anatomical structure information of soft tissues contrast with high spatial resolution. MRI has been recommended by the Gynecological (GYN) GEC-ESTRO Working Group to define the GTV in cervical cancer adaptive brachytherapy (25, 26). MRI is integrated into the radiotherapy workflow. We had to assume that TVRR after EBRT is an important prognostic factor affecting survival. In the study, we reported the prognostic value of TS, TV, and TVRR before and after EBRT in locally advanced cervical cancer patients treated with IMRT (12), evaluated relevant prognostic indicators and their effects on OS, PFS, and LFFS, and used the Youden index to determine cut-off levels for continuous tumor parameters (27). With the increased adoption of MRI before brachytherapy, the role of tumor volume in predicting treatment response in cervical cancers is being increasingly investigated. TVRR acts as an independent prognostic factor, in multivariate analysis analyzed using the Cox regression model, is an independent prognostic factor among other prognostic factors (age, FIGO stage, EBRT dose, pre- and mid-RT TS and TV, TVRR, pre- and mid-RT SCC-ag level, etc.). In 2017, a multi-center study in South Korea first reported that tumor parameters, including tumor size, tumor volume, TVRR, and SCC-ag levels, measured before intracavitary radiotherapy for cervical cancer patients were prognostically significant (12). The results showed that TVRR >87% was an independent prognostic factor for OS [HR = 3.435 (1.062–11.106)], and univariate analysis of PFS also showed that TVRR had an important effect on prognosis (*P <*0. 001). Our study reported that FIGO stage above II, pre-RT TV >61.6 cm^3^ and mid-RT TV >11.38 cm^3^ are independent adverse indicators for OS. Moreover, TVRR <82.19% was an important prognostic factor affecting OS and LFFS. For PFS, TVRR was not statistically significant. TVRR is the most important predictor for survival in our study, which are consistent with previous studies. Possible explanations are differences in treatment modalities and the population of the patients enrolled. In Korean studies (12), there were 192 patients (83.1%) with stages I and II, 39 patients (16.9%) with stages III and IV, while our study had 91 patients (41.9%) with FIGO stages IIa,b, 114 patients (52.5%) with stage III, and 12 patients with stage IVa (5.6%). Our study also verified that FIGO stage >II was an important independent adverse prognostic factor affecting OS. In multivariate analysis of our study, 5-year survival of earlier stage (II) decreased from 88.7 to 75.9% of locally advanced staging (III and IVa) (HR 2.377, 95% CI: 1.091–5.182; *P*= 0.029). The proportion of locally advanced cervical cancer patients in our study was significantly higher than in the Korean study. Lee et al. reported that the volume of cervical cancer measured by MRI was greater than 3 cm^3^ before brachytherapy, which was a poor independent prognostic factor affecting PFS (*P <*0. 0001) (28). We underline that mid-RT TV >11.38 cm^3^ (*P* = 0. 034) was an independent poor indicator of OS. Nam et al. pointed out that the residual tumor (volume >0 cm^3^) at 1 month after the end of radiotherapy was a reliable predictor of the local control (*P <*0. 01) (22). In addition, our results concluded that there were differences in prognosis and OS and significance in statistics when setting 5.5 cm as the cut-off value of pre-RT TS before radiotherapy.

Previous researchers have reported that TVRR is a more sensitive and robust factor of survival than other tumor parameters measured before or after radiotherapy (10). In other studies, some researchers have found that TVRR <75% was a significant adverse prognostic factor, and the lower tumor regression rate during radiotherapy indicates the worse prognosis (9). In the study by Nam, cervical cancer patients receiving concurrent chemoradiotherapy, the authors reported that patients with TVRR ≥75% after external irradiation had a longer disease-free survival (*P* = 0. 04) (22). Moreover, Lee and team found that TVRR ≤90% was a significant adverse prognostic factor affecting PFS in cervical cancer patients receiving radical radiotherapy (14). Mayr et al. conducted studies in another way. Their results showed when the radiotherapy dose reached 45–50 Gy, tumor residual volume ratio greater than 20% was an important poor prognostic factor for local control and disease-free survival (10). There are many possible reasons for the differences between the results of studies, including differences in the study population, different strategies for radiotherapy and chemotherapy, and different methods of measuring tumor volume. In addition, we use Youden’s index as the best cut-off point from a statistical point of view, whereas other researchers set the median value as the continuous tumor reference cut-off value (29). According to our findings, TVRR is a simple biomarker of treatment response that can be obtained non-invasively using clinical MRI without contrast agent administration or additional techniques. The TVRR is an early biomarker of prognostic and treatment efficacy in advanced cervical cancer. This finding might dramatically change the course of treatment, including combining RT with surgery and consolidative chemotherapy after treatment.

However, several factors that might affect the TVRR were also worth discussing. Firstly, previous studies have identified multiple genes that may affect radiation sensitivity by mediating the process of cervical cancer cell reproduction, differentiation, and apoptosis (30–32). Liu et al. studied the protein expression of p73 in 59 cervical cancers after radiotherapy and 68 normal cervices using immunohistochemistry. The expression of p73 was found to be significantly increased in cancer samples and sensitive to radiotherapy (*P <*0.001). Their findings suggested that p73 expression was related to the regression of cervical cancer cells and may play an important role in the regulation of cellular radiosensitivity (30). Liu proved that PXN was significantly upregulated in cervical cancer, which associated with tumor stage, poor differentiation, and led to resistance to radiation (31). Secondly, some biomarkers such as serum squamous cell carcinoma antigen (SCC-ag) also affect the TVRR. Jantharapattana conducted the study, determining a correlation between the SCC-ag level and the tumor volume regression. In their study, the critical point of SCC-ag was 5.8 ng/ml and Pearson’s correlation coefficient between SCC-ag level and tumor volume was 0.524 (*P* = 0.0002). This means SCC-ag moderately correlates with tumor volume rather than a one-dimensional measurement such as tumor size (33). The other study concluded that SCC-ag levels could predict treatment response in 96% of cases (34). Thirdly, hypoxia has also been related to tumor regression during treatment (35). During EBRT, cervical cancer regression is influenced by underlying biologic processes, including cellular sensitivity to radiotherapy and proliferation. A radiobiologic model was formulated to simulate the effect on tumor regression of the surviving proportion of cells after 2 Gy (SP(2)), and compared to the pre-treatment hypoxia measurements. The tumors with a high SP(2) >0.71 were significantly more hypoxic at diagnosis (*p* = 0.02) (35). Finally, tumor regression rate related to histologic subtype in cervical cancer during CCRT. Forty-four patients had (AC/ASC) and 354 patients had enrolled in the research. The study concludes that adenocarcinoma/adenosquamous carcinoma (AC/ASC) had a relatively poorer tumor regression rate in response to EBRT than squamous cell carcinoma (SCC) (*p <*0.001). AC/ASC histology and poor tumor response to EBRT are independent prognostic factors for worse survival in cervical cancer (36). Considering the previous reports and the results, we speculated that the early treatment response reflected by TVRR may be affected by different factors. Therefore, there is a strong need to develop novel prognostic factors and radiosensitivity. This study is promising and it might provide a useful solution for future research.

There are some limitations to our study. First, this study is limited by sample size and retrospective method. These limitations might interfere with the likelihood of true findings. Second, response assessment was primarily based on using tumor volume, which can impose other uncertainties related to under evaluation of response in very small volumes and the greater inter-observer contouring variability with this parameter (37). On the other hand, using high-risk clinical target volume as a surrogate mid-RT tumor volume, which the latter volume was not documented, might cause some overlap between the groups could exist. Third, the cut-off parameters for the TVRR were arbitrarily selected. The clinical significance of TVRR and the relationship between TVRR and biological effects of irradiation are not very clear. Therefore, more prospective or fundamental studies are needed to support the current findings.

## Conclusion

We confirmed the prognostic significance of TVRR for OS and LFFS, and pre-RT TV and mid-RT TV (before and after EBRT) were independent prognostic factors for OS. Further studies are needed in the future to verify the relationship between TVRR and the biological effects of irradiation. We expect that the early assessment of prognostic factors will help formulate the optimal treatment strategy for cervical cancer patients during radiotherapy.

## Data Availability Statement

The raw data supporting the conclusions of this article will be made available by the authors, without undue reservation.

## Ethics Statement

The studies involving human participants were reviewed and approved by the Institutional Ethics Committee of Sichuan Cancer Hospital. The patients/participants provided their written informed consent to participate in this study.

## Author Contributions

CS, SL, and JL designed the study. SL, CS, and RW developed the methodology. CS, RW, MT, and WY performed the data curation. CS drafted the manuscript. CS, SW, LW, and ML conducted the statistical analysis. CS, HZ, and JZ collected the data. HZ, WY, and RW were responsible for follow-up. SL and SW revised the manuscript. GZ and PX conducted the project administration. SL and JY provided the resources. SW provided the software. CS and SL acquired the funding. All authors listed have made a substantial, direct, and intellectual contribution to the work and approved it for publication.

## Funding

This work was supported by the Excellent Youth Program Fund of Sichuan Cancer Hospital (YB2021026), the National Natural Science Foundation of China (grant No. 81900498), and the Research Project of Science and Technology Department of Sichuan Province (grant No. 2021YFH0138; 2022JDJQ0064).

## Conflict of Interest

The authors declare that the research was conducted in the absence of any commercial or financial relationships that could be construed as a potential conflict of interest.

## Publisher’s Note

All claims expressed in this article are solely those of the authors and do not necessarily represent those of their affiliated organizations, or those of the publisher, the editors and the reviewers. Any product that may be evaluated in this article, or claim that may be made by its manufacturer, is not guaranteed or endorsed by the publisher.
